# An assessment of sampling designs using SCR analyses to estimate abundance of boreal caribou

**DOI:** 10.1002/ece3.6797

**Published:** 2020-09-19

**Authors:** Samantha McFarlane, Micheline Manseau, Robin Steenweg, Dave Hervieux, Troy Hegel, Simon Slater, Paul J. Wilson

**Affiliations:** ^1^ Environmental and Life Sciences Department Trent University Peterborough Ontario Canada; ^2^ Landscape Science and Technology Division Environment and Climate Change Canada Ottawa ON Canada; ^3^ Fish and Wildlife Stewardship Branch Alberta Environment and Parks Grande Prairie AB Canada; ^4^ Canadian Wildlife Service—Pacific Region Environment and Climate Change Canada Kelowna BC Canada; ^5^ Regional Resource Management Alberta Environment and Parks Edmonton AB Canada; ^6^ Fish and Wildlife Stewardship Branch Alberta Environment and Parks Edmonton AB Canada

**Keywords:** density estimation, noninvasive genetic sampling, population estimation, precision, spatial capture–recapture, study design

## Abstract

Accurately estimating abundance is a critical component of monitoring and recovery of rare and elusive species. Spatial capture–recapture (SCR) models are an increasingly popular method for robust estimation of ecological parameters. We provide an analytical framework to assess results from empirical studies to inform SCR sampling design, using both simulated and empirical data from noninvasive genetic sampling of seven boreal caribou populations (*Rangifer tarandus caribou*), which varied in range size and estimated population density. We use simulated population data with varying levels of clustered distributions to quantify the impact of nonindependence of detections on density estimates, and empirical datasets to explore the influence of varied sampling intensity on the relative bias and precision of density estimates. Simulations revealed that clustered distributions of detections did not significantly impact relative bias or precision of density estimates. The genotyping success rate of our empirical dataset (*n* = 7,210 samples) was 95.1%, and 1,755 unique individuals were identified. Analysis of the empirical data indicated that reduced sampling intensity had a greater impact on density estimates in smaller ranges. The number of captures and spatial recaptures was strongly correlated with precision, but not absolute relative bias. The best sampling designs did not differ with estimated population density but differed between large and small ranges. We provide an efficient framework implemented in R to estimate the detection parameters required when designing SCR studies. The framework can be used when designing a monitoring program to minimize effort and cost while maximizing effectiveness, which is critical for informing wildlife management and conservation.

## Graphical abstract

We provide an analytical framework to assess results from empirical studies to inform spatial capture–recapture sampling design, using both simulated and empirical data from noninvasive genetic sampling of seven boreal caribou populations (*Rangifer tarandus caribou*). Simulations revealed that clustered distributions of detections did not significantly impact relative bias or precision of density estimates, and analysis of the empirical data indicated that reduced sampling intensity had a greater impact on density estimates in smaller ranges. The framework can be used when designing a monitoring program to minimize effort and cost while maximizing effectiveness, which is critical for informing wildlife management and conservation.

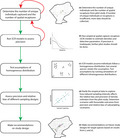

## 1 INTRODUCTION

Robust abundance estimates are fundamental parameters for managing wildlife populations, and central to understanding extinction risk (Campbell et al., 2002; Lande, 1993; Shaffer, 1981). Monitoring and understanding variation in abundance is critical for recovery efforts of threatened and endangered populations; however, producing accurate population estimates remains a challenge for many species. This is particularly true for species that occur at low density or in low abundance, that are cryptic, or that exhibit elusive behaviors which make capture difficult (Kéry, Gardner, Stoeckle, Weber, & Royle, 2011; Pollock, Marsh, Lawler, & Alldredge, 2006). Nonspatial capture–recapture (CR) analyses have been the standard method used to estimate abundance of many vertebrate species; however, spatially explicit capture–recapture (SCR) models are becoming the new standard because they are robust to small sample sizes, and can accommodate low capture probabilities (Borchers & Efford, 2008; Efford, Borchers, & Byrom, 2009; Ivan, White, & Shenk, 2013; Royle, Chandler, Sollmann, & Gardner, 2013). By including spatial information of captured individuals directly into the analyses, SCR models resolve issues surrounding the effective trapping area and are robust to assumptions about geographic closure that are common issues in nonspatial CR studies (Efford & Fewster, 2013; Royle et al., 2013). Recapturing individuals at different locations also provides information on individual activity centers, which are used to estimate animal density within the study area (Borchers & Efford, 2008; Royle et al., 2013).

SCR models directly depend on adequate number of unique individuals captured and recaptured at multiple spatial locations (Efford & Boulanger, 2019; Sun, Fuller, & Royle, 2014). Simulations are recommended to enable the assessment of sampling design on SCR parameter estimates, to inform optimal sampling design (Royle et al., 2013). Such studies have primarily focused on large carnivores, such as black bears (*Ursus americanus*; Clark, 2019; Sollmann, Gardner, & Belant, 2012; Sun et al., 2014; Wilton et al., 2014), and a few additional taxa (Kristensen & Kovach, 2018; Tobler & Powell, 2013), while limited work has been done on species occurring at low densities over large areas and with more limited home range sizes. Noninvasive genetic sampling approaches can be used to alleviate the challenges associated with surveying rare and elusive species, by constructing capture histories from DNA collected from feces, hair, or other noninvasively collected samples (Kristensen & Kovach, 2018; Lampa, Henle, Klenke, Hoehn, & Gruber, 2013; Waits & Paetkau, 2005). Noninvasive methods often result in higher capture rates and lower expense than traditional capture–recapture methods (Lampa et al., 2013; Prugh, Ritland, Arthur, & Krebs, 2005; Waits & Paetkau, 2005), and SCR is increasingly being used in combination with noninvasive methods (Kristensen & Kovach, 2018; Lamb et al., 2018; Royle et al., 2013). Knowledge of the target species’ home range size helps inform the spatial sampling design, providing reference values for the baseline detection probability (Sollmann et al., 2012; Sun et al., 2014). Efford and Boulanger (2019) presented formulae to determine the precision of new study designs by computing the expected number of detected individuals and expected number of recaptures that strongly correlate with precision. However, these formulae require reference values for density and detection parameters (Efford, 2019b), which may not be available for less studied species.

**FIGURE 1:**
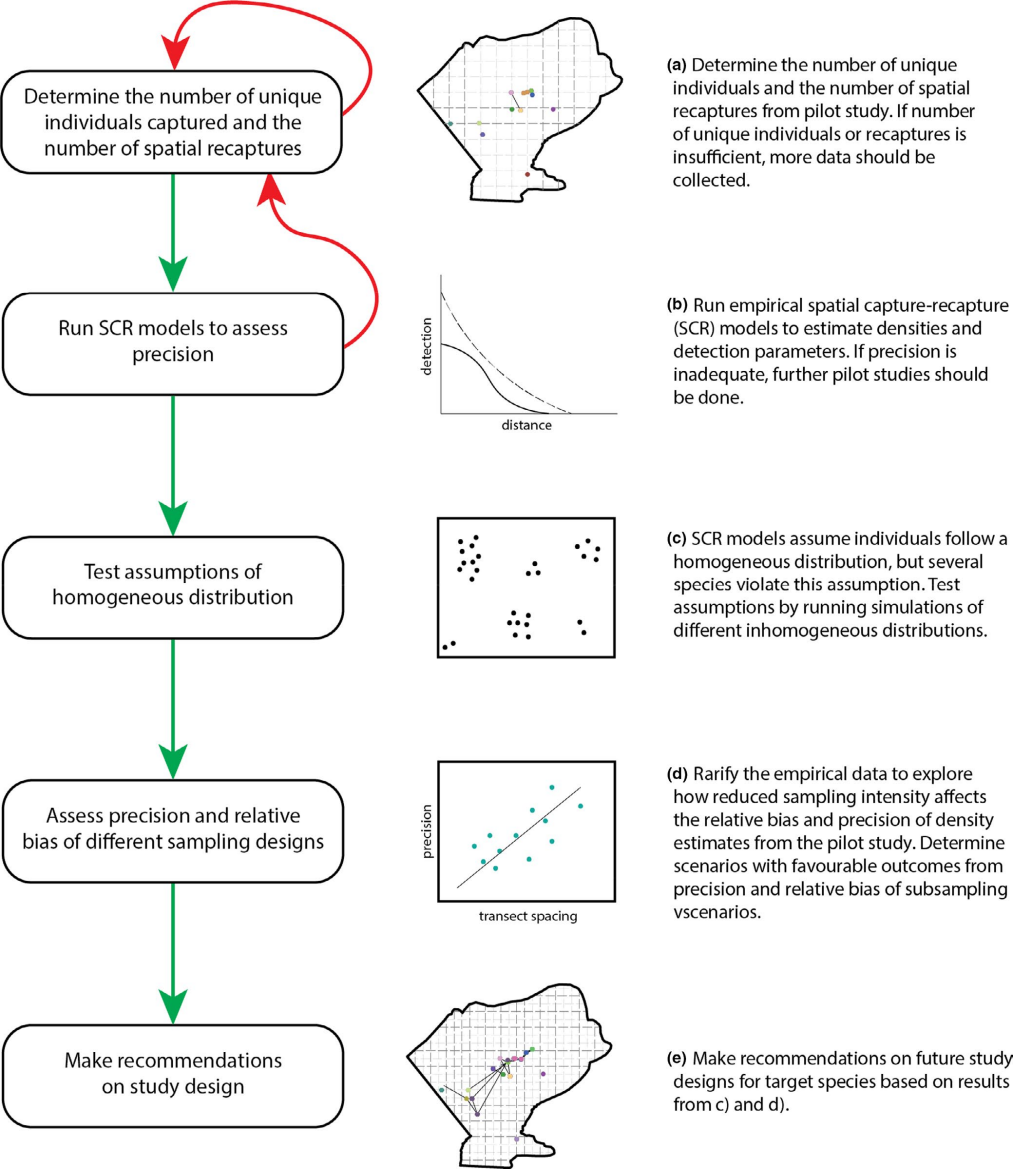
Framework for assessing results from empirical SCR studies and informing sampling designs

Here, we developed a framework to assess results from empirical studies to inform sampling designs (Figure 1). The framework consists of (1) determining the number of unique individuals captured and spatially recaptured from empirical data; (2) fitting SCR models under the assumption of homogeneous distribution to estimate the detection parameters *g0* (detection probability) and *σ* (spatial extent of an individual's use of the landscape) to assess the precision of the density estimates; (3) running simulations to assess the influence of the species’ behavior on density estimates and relative bias; (4) using empirical data to assess different sampling designs and assess precision and relative bias of the estimates; and (5) making recommendations on study design based on the resulting precision and relative bias of the estimates. The framework is implemented in R (R Core Team, 2019), using maximum likelihood methods.

**FIGURE 2:**
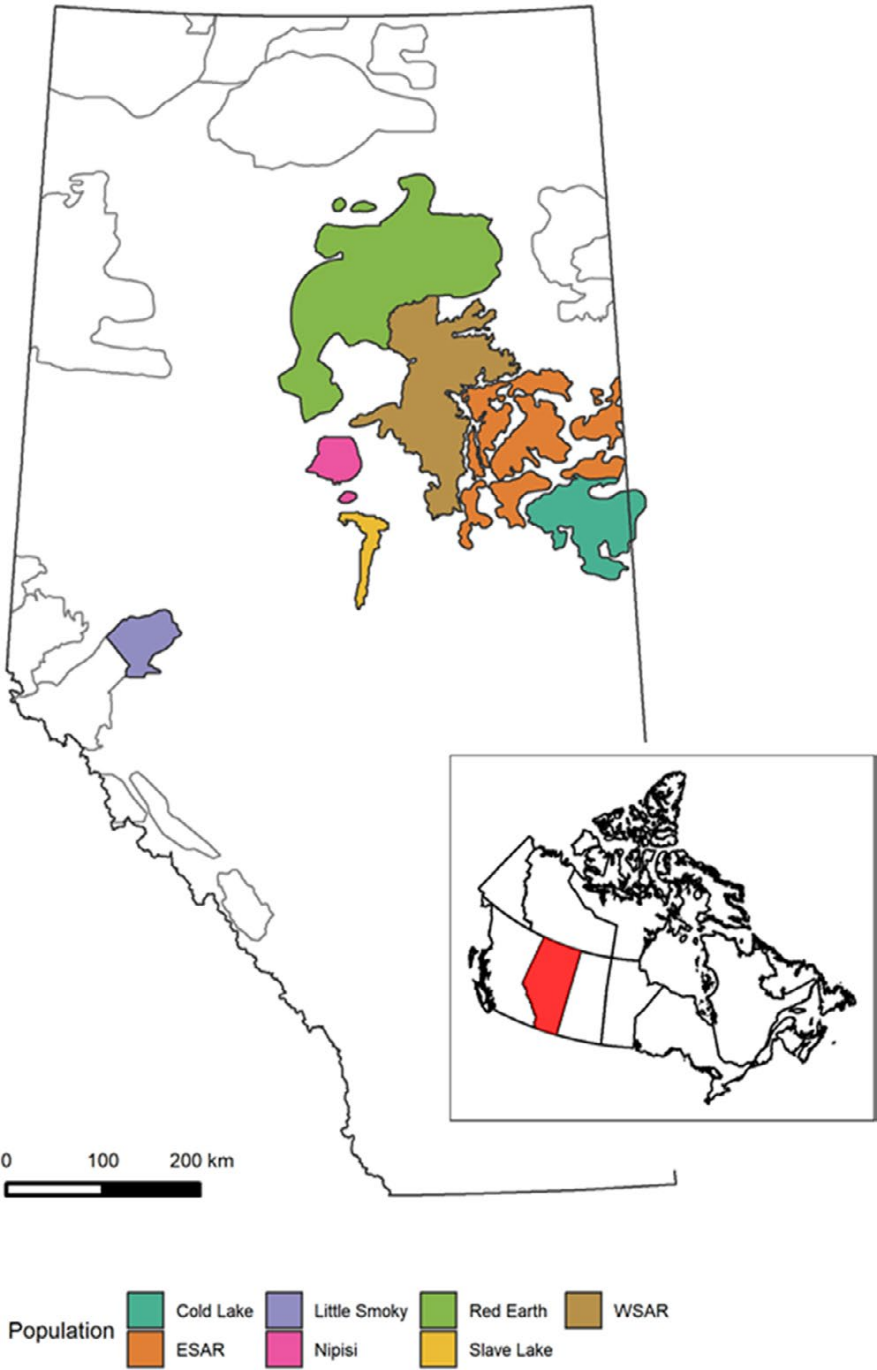
Seven boreal caribou population ranges in Alberta sampled for fecal DNA. Gray shade lines delineate other woodland caribou population ranges

To collect empirical data, we completed aerial surveys across the ranges of seven boreal caribou populations in Alberta, Canada. These ranges varied in size, exhibited differences in estimated caribou population density, and contained different levels of natural and anthropogenic disturbances (Figure 2; see Appendix S1 for details). For each caribou population, we used an aerial transect survey design to conduct noninvasive genetic sampling, through the collection of caribou fecal pellets. While we studied boreal caribou, our approach for assessing study design is applicable to other species and systems.

## 2 MATERIALS AND METHODS

### 2.1 Fecal pellet collection and genetic analysis

For each population, we flew 3 surveys to collect fecal pellets during winter (December to March), with sampling occasions spaced approximately one month apart. Following the aerial survey protocol outlined in Hettinga et al. (2012), aerial transects were systematically flown at 3‐km intervals across each entire caribou population range using rotary‐ or fixed‐wing aircraft, or a combination of both aircraft, to locate caribou feeding locations, for a total of 69,070 km flown across the seven ranges (Table 1). Once located, personnel landed at each feeding site and collected fecal samples; this included collecting samples from backtracking on caribou trails. At each feeding site, approximately 1.4 times more samples than the number of boreal caribou thought to have been present were collected to allow for a balance between capturing most individuals at a site and not recapturing the same individuals too many times. All pellet samples were kept frozen at −20°C until DNA extraction was performed.

**Table 1:** Sampling data

	Survey Year	Area Surveyed (km^2^)	Distance Flown (km)	Number of Samples Collected	Number of Samples Successfully Scored	Number of Unique Genotypes	Genotyping Success (%)	Number of Spatial Recaptures
Cold Lake	2014	7,277	7,497	844	781	261	92.5	148
ESAR	2013	13,160	13,121	1,382	1,254	401	90.7	188
Little Smoky	2014–2015	3,084	3,048	855	835	108	97.7	36
Nipisi	2018	2,104	2,119	417	415	67	99.5	72
Red Earth	2017	24,737	25,377	1,819	1,777	386	97.7	530
Slave Lake	2018	1,516	1,501	206	190	42	92.2	38
WSAR	2015	15,726	16,407	1,687	1,613	490	95.6	314
Total	‐	67,604	69,070	7,210	6,865	1,755	‐	1,326

In the laboratory, fecal samples were thawed and the mucosal coat surrounding the pellets was removed for DNA analysis. The extraction protocol used to amplify the DNA is outlined in Ball et al. (2007). Following quantification of target caribou DNA, samples were diluted down to a working stock concentration of 2.5ng/ul. We amplified the DNA at 9 variable fluorescently labeled microsatellite loci (FCB193, RT7, RT1, NVHRT16, BM888, RT5, RT24, RT6, OHEQ; Bishop et al., 1994; Cronin, MacNeil, & Patton, 2005; Wilson, Strobeck, Wu, & Coffin, 1997) to generate individual‐specific genetic profiles, along with caribou‐specific Zfx/Zfy primers for sex identification. The amplification protocol is outlined in Ball et al. (2007). Following amplification, each sample was genotyped on the ABI 3,730 DNA Analyzer (Applied Biosystems). Microsatellite alleles were scored with the program GeneMarker v1.91® (SoftGenetics, State College, PA) and followed a protocol documented in Flasko et al. (2017) and McFarlane et al. (2018). Unique individuals were identified using the program ALLELEMATCH (Galpern, Manseau, Hettinga, Smith, & Wilson, 2012). We retained samples that amplified at ≥ 5 loci and re‐amplified apparent unique genetic profiles represented by a single sample using two independent scorers to confirm unique individual identities (Hettinga et al., 2012). An error rate per locus was calculated using these re‐amplification results.

### 2.2 Framework

#### 2.2.1 Empirical SCR modeling

We used a maximum likelihood approach implemented in the R package *secr* (Efford, 2018; R Core Team, 2019) to estimate boreal caribou densities. SCR models are comprised of a submodel for the distribution of animals in the area of study (population density, *D*), and a submodel for the detection process, given the detection probability (the intercept of the detection function, *g0*) and given a parameter for scaling the detection function (the spatial extent of an individual's use of the landscape—*σ*Borchers & Efford, 2008; Efford et al., 2009). For our empirical data, we treated each survey as an occasion within a single session. We discretized the study area into a 1,500 m grid of proximity detectors (which record the presence of individuals at each detector without restricting movement; Efford et al., 2009), and each grid was sampled in each occasion with the same search intensity. The area of integration for SCR models needs to be large enough such that animals residing beyond the study area have a negligible chance of being detected (Borchers & Efford, 2008; Efford, 2004; Royle & Young, 2008). We therefore defined our state‐space with a 15‐km^2^ buffer around all study areas. We ran models for females, males, and both females and males together.

We estimated the parameters of the SCR detection function (*g0* and *σ*) by maximizing the conditional likelihood, and derived density (*D*) from the top AIC_C_‐ranked models (Anderson, Burnham, & White, 1994; Borchers & Efford, 2008). We used the hazard exponential form of the detection function, because area search data models the cumulative hazard of detection (Efford, 2011). Models assumed that individuals were identified correctly, populations were demographically closed during sampling, and detections were independent and conditional on activity center (Borchers & Efford, 2008; Efford, 2004). We assessed sources of variation on the detection parameters with time and behavior effects on both *g0* and *σ*.

#### 2.2.2 Testing assumptions of homogeneous distribution

Boreal caribou is a nonmigratory ecotype of caribou and have relatively small home ranges compared to wide‐ranging carnivores such as brown bears (Graham & Stenhouse, 2014; Lamb et al., 2018) and black bears (Whittington & Sawaya, 2015). Boreal caribou exhibit a fission‐fusion social structure and dynamics, with group size fluctuating throughout the year and frequent exchanges between groups; group size is lowest during spring and summer when cows become solitary for calving, increases before the rut, and may increase or decrease during the winter (Thomas & Gray, 2002). To assess how the distribution of the animals (i.e., clustering) affected the precision and relative bias of our estimates, we simulated different population distributions at the individual level using three of our empirical datasets (Little Smoky, Cold Lake, and Slave Lake). Different distributions can be used for the simulations including a homogeneous Poisson distribution, inhomogeneous, or clustered Poisson distributions (Efford, 2019a). The chosen population distribution should reflect the distribution of the study species. Our empirical data approximated a Neyman‐Scott clustered Poisson distribution which was then used for the simulations (Efford, 2019a). To simulate multiple detections in very close proximity, we set the spatial scale (*σ*) of the 2D kernel for locations within each cluster to be 1. To simulate varying levels of clustering, we varied the fixed number of individuals per cluster (see Appendix S2: Figs S2.1–S2.3). We selected starting values for D, *g0,* and *σ* from the empirical model runs (Table 2). We carried out all simulations in the secr R package (Efford, 2018; R Core Team, 2019).

**Table 2:** Spatially‐explicit capture–recapture density estimates for boreal caribou in Alberta, Canada. Density estimates (D) are per 1,000 km^2^, SE(D) is the standard error of the density estimate, CV(D) is the coefficient of variation (SE of density estimate/density estimate), g0 indicates the capture probability at the home range center, sigma is the spatial scale parameter in meters, and N is the abundance over the study area

	D (95% CI)	SE(D)	CV(D)	g0 (95% CI)	*σ* (95% CI)	*N* (95% CI)
Cold Lake	61.9 (46.3–82.9)	6.69	0.15	0.015 (0.007–0.031)	3,363.2 (2,215.1–5240.1)	353 (276–452)
ESAR	50.6 (42.9–59.6)	4.24	0.08	0.024 (0.015–0.039)	1778.8 (1,451.8–2180.5)	647 (549–763)
Little Smoky	31.1 (22.8–42.5)	4.99	0.16	0.028 (0.006–0.124)	1603 (799.6–3213.9)	94 (69–129)
Nipisi	30.7 (22.8–41.4)	4.70	0.15	0.053 (0.027–0.104)	1941.6 (1,419.6–2658.9)	63 (47–85)
Red Earth	16.1 (14.4–17.9)	0.87	0.05	0.022 (0.019–0.026)	3,124.8 (2,935.3–3326.5)	387 (347–430)
Slave Lake	25.9 (17.2–39.1)	5.51	0.21	0.247 (0.061–1.023)	1,226 (772.4–1952.3)	38 (25–58)
WSAR	43 (38.5–48.1)	2.43	0.06	0.013 (0.011–0.016)	2,868.9 (2,701.5–3046.6)	659 (590–737)

#### 2.2.3 Assessing precision and relative bias of different sampling designs using empirical data

We repeated the empirical population analyses with subsamples of data to explore how reduced sampling intensity affected the relative bias and precision of the density estimates from our empirical study. We rarified the data by reducing the number of sampling occasions and reducing the number of aerial transects flown. For the reduced number of sampling occasions, all possible 2‐occasion combinations were run (occasions 1 and 2; occasions 2 and 3; and occasions 1 and 3). Aerial transects were removed from the original spatial field data, keeping either every second or third transect line to emulate sampling strategies of 6 km or 9 km transects. Only the samples collected along the remaining transect lines were retained, and only those detectors along the remaining transect lines were used in the analysis. We used the coefficient of variation (CV) as the metric for precision, and calculated the absolute relative bias (|*RB| =* |*(* $\hat{D}$ *‐D)/D*|) as the metric for bias (as in Efford & Boulanger, 2019; Efford & Fewster, 2013; Kristensen & Kovach, 2018; Tobler & Powell, 2013). We compared estimates from the reduced datasets ($\hat{D}$) to those based on the empirical dataset (*D*). We considered models with CV < 20% (following Pollock, Nichols, Brownie, & Hines, 1990) and relative bias < 15% (Otis, Burnham, White, & Anderson, 1978) as favorable outcomes. Models with CV < 30% and |RB| <20% can also be considered favorable (Kristensen & Kovach, 2018), because high precision may be difficult to achieve for rare and low‐density species.

We calculated the precision and relative bias of each subsampling scenario. To determine how the number of captures, number of recaptures, and number of spatial recaptures (recaptures at different locations) influence the precision and relative bias of the estimates, we correlated the precision and relative bias of the estimates with these parameters for each scenario, and then globally.

## 3 RESULTS

### 3.1 Capture and spatial recapture rates

A total of 7,210 fecal samples were collected and 6,865 were successfully genotyped (average 95.1% genotyping success), resulting in the identification of 1,755 unique individuals from the seven populations detected a total of 1,326 times (unique site‐occasion‐animal detections (spatial recaptures); Table 1). Only four allelic dropout amplification errors occurred (error rate < 0.001%). The number of captures (*n* = 85–931) varied with range size, and proportion of captures that were recaptured (34%–58%), and spatially recaptured (31%–57%) was highest in Red Earth and lowest in ESAR (Appendix S3: Table S3.1, Table 1). We had similar recapture and spatial recapture rates for females and males (Appendix S3: Tables S3.2, S3.4).

### 3.2 Empirical model performance

Density estimates for the seven populations ranged from 16.1 to 61.9 caribou/1,000 km^2^ (Table 2). The coefficient of variation varied from 5% to 21% for both sexes combined, from 7% to 22% for females, and from 8% to 54% for males (Table 2, Appendix S3: Tables S3.3, S3.5). The average detection probability was low (*g0* < 0.06; Table 2) for all populations except the first sampling occasion for Slave Lake (*g0_t1_* = 0.66, *g0_t2_* = 0.036, *g0_t3_* = 0.44). *σ* differed among populations, ranging from 1,226 m in Slave Lake to 3,363 m in Cold Lake (Table 2).

### 3.3 Assumptions of homogeneous distribution

Results of simulations showed that clustering of caribou detections did not impact the precision or relative bias of the density estimates (Appendix S2). Median density estimates remained similar and slightly above the starting density for all levels of clustering density (*µ*) for the three simulated populations. The simulated Cold Lake population estimates retained the highest precision and were relatively unbiased, despite clustering, which corresponds with the precision found for the empirical model (Table 2). The simulated Little Smoky and Slave Lake population density estimates had lower precision than Cold Lake when caribou were clustered, but median density estimates were not affected by clustering, and density estimates from both populations remained unbiased (Appendix S2). Using a threshold value for precision of CV < 20%, Little Smoky and Slave Lake had inadequate median levels of precision at all levels of *µ*. These populations had similar (Little Smoky = σ1,600 m) or smaller (Slave Lake = σ1,200 m) σ values compared to the chosen detector spacing of 1,500 m (see Appendix S4). The detector spacing of 1,500 m for the empirical studies for these populations was too wide relative to σ, with very few spatial recaptures of individuals (36 in Little Smoky, 38 in Slave Lake over three occasions), as the detector spacing was larger than σ.

### 3.4 Precision and relative bias of reduced sampling designs

**FIGURE 3:**
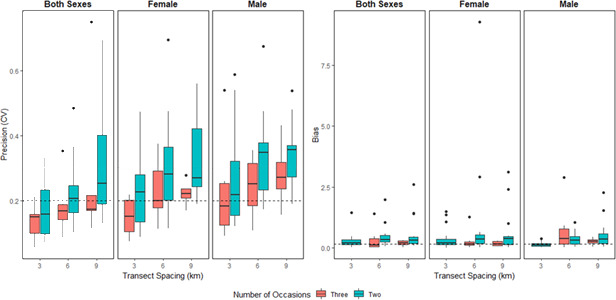
Measures of precision (CV) and bias (absolute relative bias, |RB|) for boreal caribou density estimates from subsampled empirical data (two or three sampling occasions, transect spacings of 3, 6, and 9 km) for both sexes, females and males. Dashed lines for CV represent 20% and 30% CV, and the dashed lines on RB represent 15% RB. Note: some outliers were dropped for data display

**FIGURE 4:**
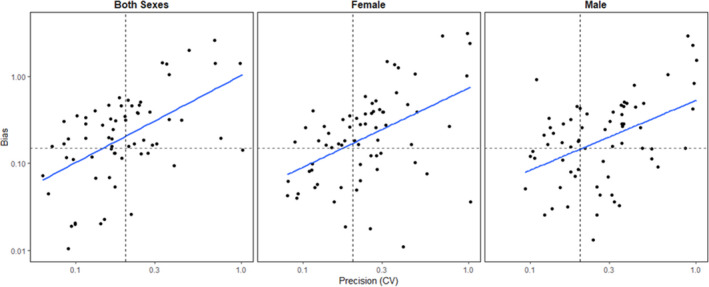
Relationship between absolute relative bias (|RB|) and precision (Coefficient of Variation) for boreal caribou density estimates from subsampled empirical data (two or three sampling occasions, transect spacings of 3, 6, and 9 km) for both sexes, females and males. Dashed lines for CV represent 20% and 30% CV, and the dashes lined on RB represent 15% RB

In total, 36 different subsampling scenarios were run for each population, for a total of 252 models. Precision and relative bias were positively correlated for all sexes (both sexes *r* = 0.557, *p* < .0001, female *r* = 0.597, *p* < .0001, male *r* = 0.634, *p* < .0001). Precision decreased (increased CV) and relative bias increased (divergence from the estimate from the full dataset) with increased transect spacing and reduced number of occasions (Figures 3, 4). Several scenarios failed to converge for Little Smoky and Slave Lake at the reduced 6 km and 9 km transects due to low numbers of individuals and no recaptures, resulting in 227 completed models. The Little Smoky and Slave Lake ranges are two of the geographically smallest ranges (Table 1; Figure 2), and samples in these areas were clustered geographically (Figure 2). The detection function scaling parameter (σ) for the empirical data for Little Smoky and Cold Lake were smaller than the detector spacing of 1,500 m and reducing the number of transects increased the detector spacing even further, leading to the detector spacing being significantly larger than the σ estimates for these populations.

**FIGURE 5:**
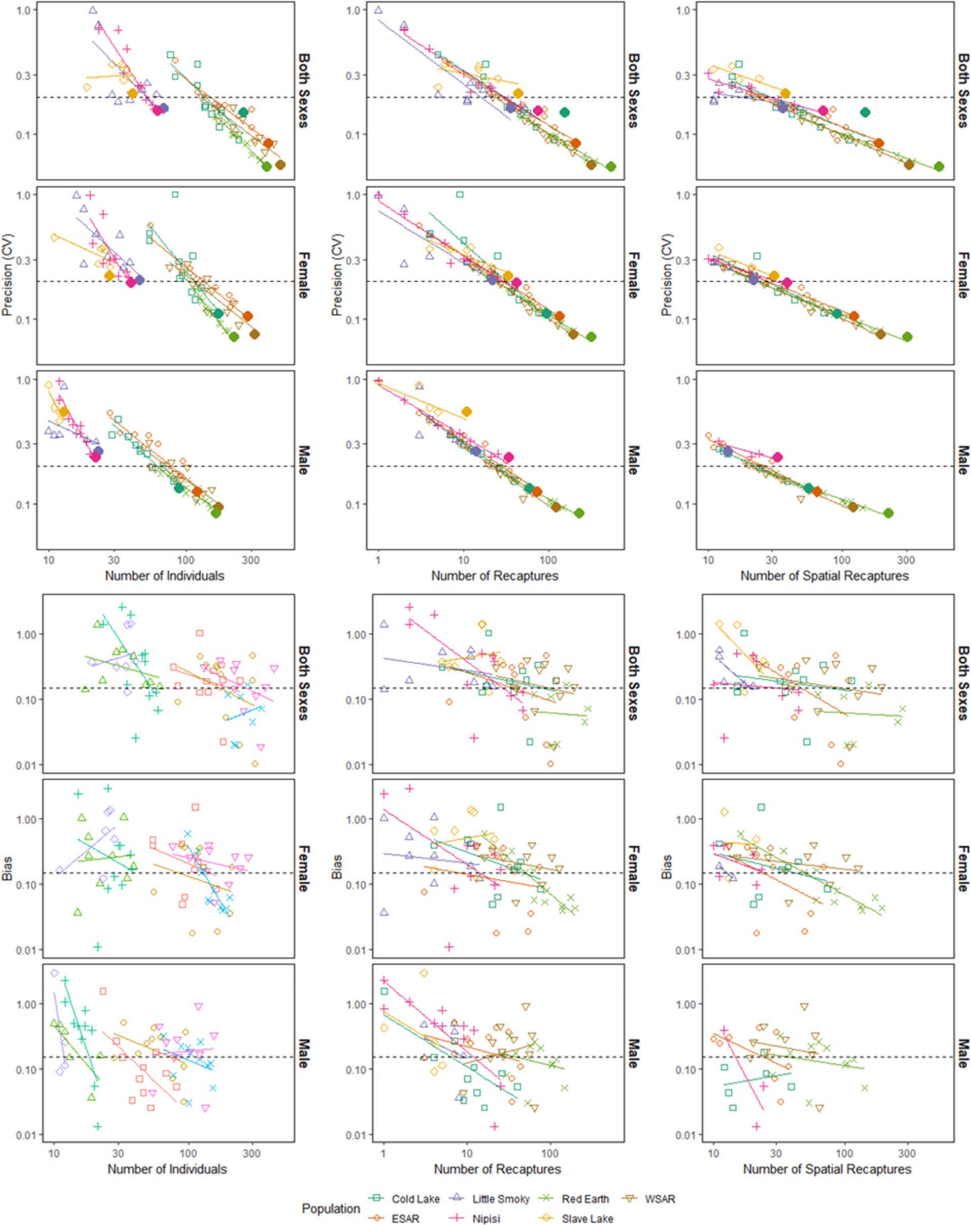
The relationship between the number of captures and recaptures and the precision (CV) and bias (absolute relative bias, |RB|) of density estimates for 7 populations of boreal caribou from subsampled empirical data (two or three sampling occasions, transect spacings of 3, 6, and 9 km) for both sexes, females and males. For each population, fewer unique individuals are sampled as the data are rarified to simulate decreasing sampling intensities, with filled circles indicating the full empirical datasets. Dashed lines for CV represent 20% and 30% CV, and the dashed lines on bias represent 15% bias

Precision of the subsampling scenarios was influenced by the number of unique individuals, number of recaptures, and number of spatial recaptures (Figure 5). Precision was negatively correlated with the number of individuals, with precision decreasing with fewer captured individuals (Appendix S3: Table S3.6, Figure 5); all models that failed to run had no recaptures of individuals. The larger ranges of Cold Lake, ESAR, WSAR, and Red Earth had more unique individuals than the smaller ranges of Little Smoky, Nipisi, and Slave Lake (Figure 5). When determining the influence of the number of individuals on model precision, all models with three occasions had adequate precision (<20% CV) for both sexes in the larger populations. The number of unique individuals had a greater influence in the smaller ranges, leading to inadequate precision in Little Smoky, Nipisi, and Slave Lake (Figure 5), with no significant correlation between precision and the number of unique individuals in Slave Lake (both sexes) and Little Smoky males (Appendix S3: Table S3.6). CV was negatively correlated with the number of recaptures (Appendix S3: Table S3.7) and spatial recaptures (Appendix S3: Table S3.8), with lower precision in the smaller populations compared to the larger populations. All models with three occasions for the larger populations fell below the 20% CV threshold for all sex models (Figure 5). Even when decreasing the number of occasions to two, the larger ranges still performed well with adequate precision, as these subsets still provided an adequate number of recaptures of individuals for the models to run and precision was significantly correlated to the number of recaptures (Appendix S3: Table S3.7, Figure 5). The smaller ranges did not perform as well when the data were reduced to two occasions; several models only retained one recapture of an individual, which resulted in a CV of nearly 100% (Figure 5), and the number of recaptures or spatial recaptures was not significantly correlated with precision (Slave Lake both sexes, Little Smoky males, Slave Lake males; Appendix S3: Table S3.7‐Table S3.8).

**FIGURE 6:**
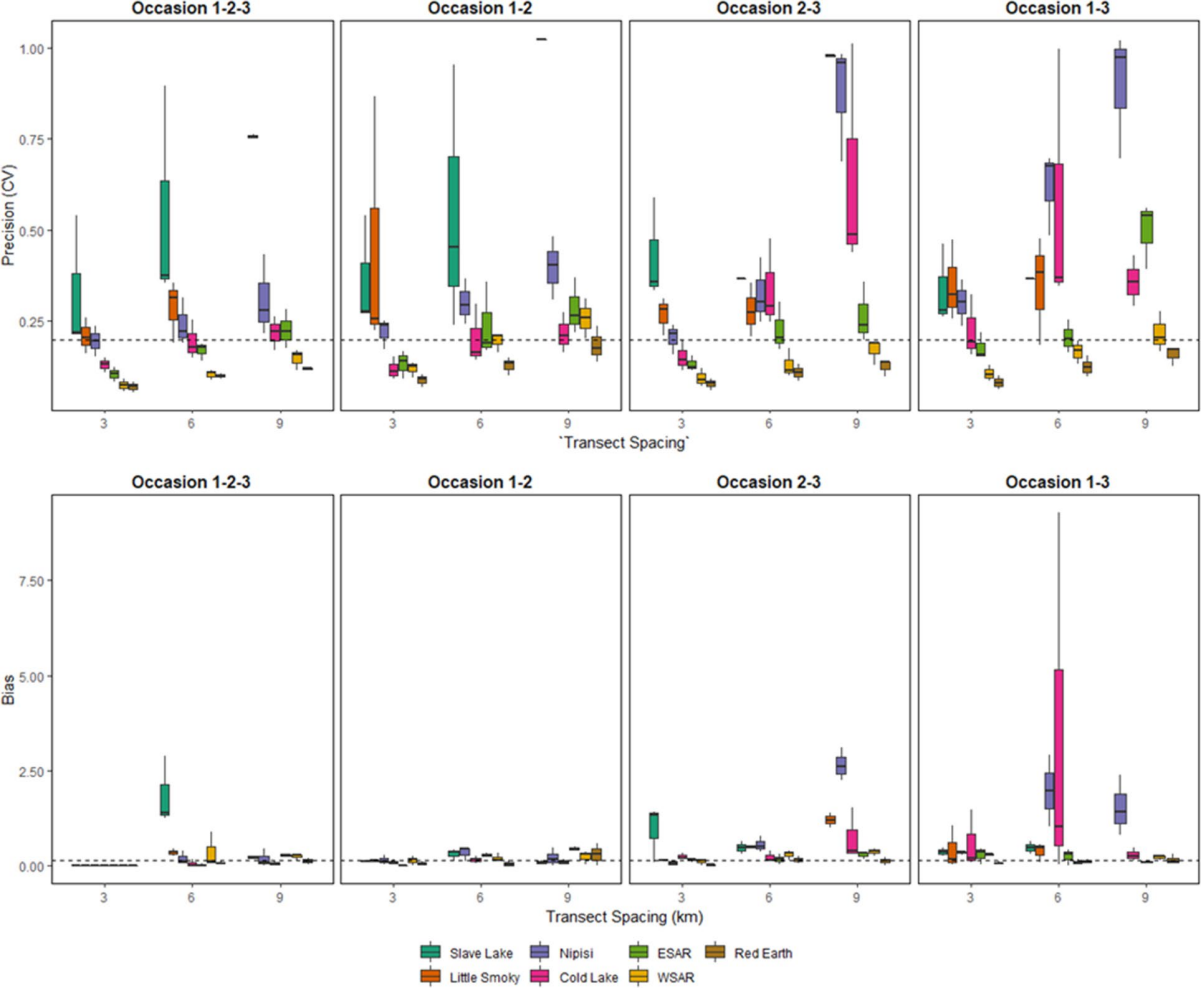
Measures of precision (CV) and bias (absolute relative bias, |RB|) boreal caribou density estimates from subsampled empirical data (two or three sampling occasions, transect spacings of 3, 6, and 9 km) for both sexes, females and males in each range. Note that some bias values were not displayed on the graph as they were extremely inflated

While there was a strong relationship between precision and the number of individuals and recaptures, this was not the case for relative bias (Appendix S3: Tables S3.6–S3.8; Figure 5). Except for Nipisi (all sexes) and Red Earth females, the number of captures, number of unique individuals, recaptures, or spatial recaptures was not significantly correlated with relative bias (Appendix S3: Tables S3.6–S3.8). Removing the third session resulted in more bias compared to removing the first and second sessions (Figure 6).

## 4 DISCUSSION

We provide an efficient framework for estimating detection parameters required for SCR studies and assessing empirical study designs for species where baseline detection data is not available. Our results using seven empirical datasets indicate that our genotyping protocol was highly successful, our capture and recapture rates were sufficient, and our study design was appropriate in producing precise and reliable density estimates. We followed the aerial survey protocol outlined in Hettinga et al. (2012) to inform our sampling design and obtained similar recapture rates between sampling occasions. We found that the detection parameters *g0* (detection probability) and σ (the spatial extent of an individual's use of the landscape) varied among our study populations and between sexes (Table 2, Appendix S3: Tables S3.3, S3.5). Our results were robust to reduced sampling intensity (both in frequency and spatially), with the best study design dependent upon range size, and not dependent upon estimated population density or the spatial distribution of individuals.

For multiple species, the SCR model assumption that animals are independently and uniformly distributed over a study area is often violated, as is the case for boreal caribou (Després‐Einspenner, Howe, Drapeau, & Kuhl, 2017; López‐Bao et al., 2018; Stevenson et al., 2015). The fission–fusion social structure and dynamics exhibited by boreal caribou during the winter months leads to frequent exchanges between small, dynamic groups (Thomas & Gray, 2002). Our simulation results show that SCR models performed reliably; the grouping and movement patterns of boreal caribou during our sampling period had minimal impact on the precision or relative bias of the density estimates. Density estimates from the simulations were estimated slightly high (Appendix S2) across all clustering levels, but the source of bias was not related to the clustering simulations, as the precision and relative bias remained consistent when varying the level of clustering. Few studies have looked at the effect that nonindependence of individuals has on SCR methodologies. López‐Bao et al. (2018) simulated scenarios of nonindependence and spatial aggregation of individual wolves (*Canis lupus*) with only a slight underestimation in population abundance estimates of aggregated individuals, while Després‐Einspenner et al. (2017) were unsure to what extent the measures of uncertainty in their study of a community western chimpanzees (*Pan troglodytes verus*) were underestimated. Bischof, Dupont, Milleret, Chipperfield, and Royle (2020) found that SCR models are robust to moderate levels of aggregation and cohesion, with low to moderate aggregation and cohesion not impacting the bias and precision of density and σ estimates. Inferences from SCR density estimates for species with small group sizes can be trusted even if grouping is ignored (Bischof et al., 2020). Although the fission–fusion social structure of caribou leads to frequent exchanges of individuals between groups, boreal caribou were rarely resampled together as a group or as a pair in our study (unpublished data).

Study designs can be inappropriate when poorly matched with the spatial behavior of the target species (Williams, Nichols, & Conroy, 2002). Detector arrays that are significantly smaller than one home range, or extreme detector spacing that leads to few or no spatial recaptures can result in biased SCR estimates (Efford, 2011; Efford & Boulanger, 2019; Sollmann et al., 2012; Tobler & Powell, 2013). Reducing the sampling intensity had a greater impact on populations with smaller range sizes regardless of density; reducing the number of transects flown led to extreme detector spacing with few or no spatial recaptures. Increasing the temporal period of sampling or decreasing the width between transects flown can be an effective way of increasing the number of detected captures and recaptures available for analysis, which increases precision; however, increasing the temporal sampling period can also violate the assumption of population closure and lead to biased estimates (Dupont, Milleret, Gimenez, & Bischof, 2019). We found that the effects of reducing the number of sampling occasions on density estimates was influenced by the timing of the survey. If resources were only available to perform 2, rather than 3, sampling sessions, we recommend focusing on collecting samples early in the winter, rather than later in the winter, as we achieved relatively unbiased estimates (|RB| <20%) when retaining December, January, or February sampling occasions. Weather conditions during March surveys were not always favorable, with poor snow conditions and warm temperatures creating difficulties for finding animals and identifying fresh tracks and feeding areas.

Results from our empirical study provides a range of estimates that can be used for simulating surveys of boreal caribou in other locations. For poorly studied species, completing an initial empirical study is critical for obtaining accurate detection probability estimates. Due to the clustered, nonhomogeneous distribution of boreal caribou, extensive sampling of the entire population is recommended to ensure that clusters of caribou are not missed during sampling. Our subsampling scenarios showed how less extensive sampling in smaller ranges can miss a large portion of the population, increasing the relative bias and imprecision of the density estimates. Applying the same sampling design to all seven of our study populations proved to be suboptimal; detector spacing for the smaller populations relative to sigma led to imprecise estimates. Our subsampling scenarios were systematically done by reducing the sampling effort through reduced detectors, occasions, or a combination of both. Our study system was extensive, with large and spatially representative sample sizes, leading to 252 models used in assessing the precision and bias of our reduced sampling scenarios. We advocate that researchers with smaller study systems use multiple subsets and averages where meaningful.

Our analytical framework allowed us to examine the results of empirical surveys in depth, providing confidence in the density estimates. Through different simulations we were able to explore how relative bias and precision of estimates vary when assumptions are violated. We showed that the number of individuals and recaptures of individuals can be used to predict precision, but that they cannot be used to predict relative bias. Efford and Boulanger (2019) state that subsampling of data to emulate different configurations of detectors, or different temporal sampling can be prohibitively slow, due to model fitting being computer‐intensive; however, we found that even for our largest population model (24,737 km^2^, 386 unique individuals, and 545 recaptures), modeling with time and behavior effects on both *g0* and σ ran relatively quickly (~7–10 days on a high‐performance computer cluster) in a maximum likelihood framework, where the density model was fitted by maximizing the conditional likelihood.

We recommend the combination of noninvasive DNA sampling, together with SCR modeling and distribution simulations, to be an effective, accurate and precise approach to monitoring wildlife.

## CONFLICT OF INTEREST

None declared.

## AUTHOR CONTRIBUTION

**Samantha McFarlane:** Formal analysis (lead); Methodology (equal); Software (equal); Validation (lead); Visualization (lead); Writing‐original draft (lead); Writing‐review & editing (lead). **Micheline Manseau:** Conceptualization (equal); Formal analysis (supporting); Funding acquisition (equal); Investigation (equal); Methodology (lead); Project administration (lead); Supervision (equal); Writing‐original draft (supporting); Writing‐review & editing (lead). **Robin Steenweg:** Conceptualization (equal); Data curation (equal); Formal analysis (supporting); Funding acquisition (equal); Methodology (supporting); Project administration (equal); Software (supporting); Validation (supporting); Writing‐review & editing (supporting). **Dave Hervieux:** Conceptualization (equal); Data curation (equal); Funding acquisition (equal); Project administration (equal); Writing‐review & editing (supporting). **Troy Hegel:** Conceptualization (equal); Data curation (supporting); Writing‐review & editing (supporting). **Simon Slater:** Conceptualization (equal); Data curation (equal); Writing‐review & editing (supporting). **Paul Wilson:** Data curation (equal); Methodology (supporting); Project administration (equal); Supervision (equal); Writing‐review & editing (supporting).

### OPEN RESEARCH BADGES

This article has earned an Open Data Badge for making publicly available the digitally‐shareable data necessary to reproduce the reported results. The data is available at https://doi.org/10.5061/dryad.v9s4mw6st.

## Supporting information

Appendix S1‐S4

## Data Availability

Data and R scripts deposited in the Dryad Digital Repository (https://doi.org/10.5061/dryad.v9s4mw6st).
