# Supplementary material for: An assessment of sampling designs using SCR analyses to estimate abundance of boreal caribou
Source: Ecol Evol. 2020 Sep 19;10(20):11631–42. doi: 10.1002/ece3.6797 (PMC7593142; doi:10.1002/ece3.6797)
Supplement: Supplementary file 1 — Appendix S1‐S4 [file ECE3-10-11631-s001.docx]

##### Appendix 1: Caribou ranges and sampling protocols

Woodland caribou (*Rangifer tarandus caribou*) are a wide-ranging deer species found throughout Canada’s boreal forests, and face increasingly severe threats across their range from habitat loss, habitat fragmentation, and increased predation from landscape changes; populations have steadily decreased throughout most of their range, with the southern limit of boreal caribou distribution receding northward since the 1990’s (COSEWIC, 2002; Environment Canada, 2011; Festa-Bianchet, Ray, Boutin, Côté, & Gunn, 2011; Hervieux et al., 2013; Schaefer, 2003). Boreal caribou require large areas of undisturbed, continuous habitat, preferring mature to old-growth coniferous forests abundant with lichens, or peat lands and muskegs along with upland and hilly areas (Courtois, Bernatchez, Ouellet, & Breton, 2003; Environment Canada, 2012; Rettie & Messier, 2000; Stuart-Smith, Bradshaw, Boutin, Hebert, & Rippin, 1997). Both natural and anthropogenic disturbances affect the spatial distribution and habitat selection of boreal caribou. Maintaining low densities is a key behavioural pattern for boreal caribou to avoid predation and ensure adequate recruitment rate and adult survival, but altered habitats from human land uses are no longer optimal for boreal caribou (e.g. Smith, Ficht, Hobson, Sorensen, & Hervieux, 2000).

Boreal caribou present a good opportunity for evaluating optimal sampling design for non-invasive SCR studies. Boreal caribou are a non-migratory ecotype of caribou and have relatively small home ranges compared to wide-ranging carnivores such as brown bears (Graham & Stenhouse, 2014; Lamb et al., 2018) and black bears (Whittington & Sawaya, 2015), for which most prior SCR sampling design studies have been performed (Sun et al., 2014; Wilton et al., 2014). Boreal caribou are habitat specialists, and maintain low population densities as an anti-predator strategy (Environment Canada, 2012; Rettie & Messier, 2001), adding further challenges to abundance estimation. We assessed seven populations of boreal caribou from Alberta, Canada: Cold Lake, Little Smoky, East Side Athabasca River (hereafter referred to as ESAR), West Side Athabasca River (hereafter referred to as WSAR), Red Earth, Slave Lake, and Nipisi.

The Cold Lake range (6,724 km^2^) in north-eastern Alberta and adjacent areas in western Saskatchewan and shares a border with the ESAR range. 87% of the Cold Lake range is disturbed from natural and anthropogenic sources (primarily through extraction of natural resources such as oil, gas and timber), with wildfire accounting for 30% of the current disturbance within the range (Government of Alberta, 2017). Seismic lines, pipelines and abandoned wells make up a large legacy footprint within the range, with active restoration of seismic lines occurring in portions of the range (Government of Alberta, 2017). The Cold Lake Air Weapons Range is located within the Cold Lake caribou range boundary. The Little Smoky range (3,084 km^2^) is the last remaining boreal population of caribou occurring in the eastern slopes of west-central Alberta (Government of Alberta, 2017). 99% of the Little Smoky range is disturbed from natural and anthropogenic sources (primarily petroleum, natural gas and forestry), with wildfires accounting for less than 1% of the disturbance within the range (Government of Alberta, 2017). The ESAR range (13,160 km^2^) is located in north-eastern Alberta, comprised of seven disjuncted sub-range areas, and shares a border with the Cold Lake range. 88% of the ESAR range is disturbed from natural and anthropogenic sources (primarily forestry, oil sands, petroleum and natural gas), with wildfire accounting for 32% of the current disturbance within the range (Government of Alberta, 2017). The ESAR range is highly fragmented, with nearly 20,000 km of seismic lines and numerous wells, roads and pipelines throughout the range (Government of Alberta, 2017). The WSAR range (15,727 km^2^) is located in northern Alberta, and shares a border with the Red Earth boreal caribou population. 84% of the WSAR range is disturbed from natural and anthropogenic sources (primarily human footprint from legacy seismic lines, pipelines, wells, roads, forest harvest and transmission lines), with wildfire accounting for only 6% of the disturbance within the range (Government of Alberta, 2017). The Red Earth range (24,737 km^2^) is located in northern Alberta, and shared a border with the WSAR boreal caribou population. 68% of the Red Earth range is disturbed from natural and anthropogenic sources (primarily from forestry, oil sands and natural gas), with wildfires accounting for 38% of the total disturbance (Government of Alberta, 2017). The Slave Lake range (1,516 km^2^) is located in central Alberta. 99% of the Slave Lake range is disturbed from natural and anthropogenic sources (primarily forestry, petroleum, mineral and aggregate resource extraction), with wildfires account for 37% of the current disturbance within the range (Government of Alberta, 2017). The Nipisi range (2,104 km^2^) is located in central Alberta. 94% of the Nipisi range is disturbed from natural and anthropogenic sources (primarily forestry, oil, and gas), with wildfires accounting for only 8% of the current disturbance within the range (Government of Alberta, 2017).

**Sample collection information**

We collected 867 samples from the Little Smoky caribou range and excluded 12 non-caribou samples (putative deer) and 20 samples that were contaminated or extremely poor quality. The resulting dataset of 835 samples representing 108 unique genotypes (73 females and 35 males) amplified at ≥7 loci, including 5 profiles that occurred once within the dataset (hereafter, singletons). From the WSAR region, we collected 1,701 samples including 15 non-caribou samples (14 deer plus 1 putative moose) and 73 samples that were contaminated or extremely low quality. The resulting dataset from WSAR included 1,613 samples representing 489 unique genotypes (316 females and 173 males), 98% of which amplified at ≥7 loci, and 130 singletons. We collected 1,460 samples from the ESAR region, including 78 non-caribou samples (deer) and 122 samples that were contaminated or extremely low quality. The resulting dataset from ESAR included 1,254 samples representing 401 unique genotypes (281 females and 120 males), 92% of which amplified at ≥7 loci, and 122 singletons. From the Cold Lake region, we collected 931 samples, including 87 non-caribou samples (putative deer) and 62 samples that were contaminated or extremely low quality. The resulting dataset from Cold Lake included 781 samples representing 261 unique genotypes (172 females and 89 males), 93% of which amplified at ≥7 loci, and 87 singletons. Only four unique genotypes were seen in more than one range (3 genotypes in both ESAR and Cold Lake and 1 genotype in both ESAR and WSAR) (Hileman et al., *unpublished data*). Conditions during the March survey in Cold Lake were particularly difficult, with temperatures as high as +8°C during some afternoons, compounded by the fact that flying was only permitted in the Cold Lake Air Weapons Range during weekends; only 16 sites were visited during this survey, and ~1/3 of the number of samples were collected compared to during January and February surveys.

**Additional SCR model information**

Aerial transects were flown 3 km apart; discretizing our search data to 1500 meters represents half the width of the aerial transects, which is the distance observers can see out of each side of the helicopter. Initial exploratory analyses were completed from 750 metre to 2500 metre discretize spacing, at intervals of 250 meters (8 different discretizations in total) to determine the best spacing; discretization of 1500 meters was the best performing discretization (see Appendix 4). The area of integration for SCR models needs to be large enough that animals residing beyond the study area have a negligible chance of being detected (Efford, 2004; Borchers & Efford, 2008; Royle & Young, 2008). We therefore defined our state-space with a 15 km^2^ buffer around all study areas. Models were run with both males and females modelled together, and sexes were run separately to get sex-specific estimates.

We estimated the parameters of the SCR detection function (*g0* and $\sigma$) by maximizing the conditional likelihood. We used the hazard exponential form of the detection function, as area search data models the cumulative hazard of detection (Efford, 2011); initially, the hazard halfnormal and hazard exponential detection functions were compared via the Akaike Information Criterion adjusted for small sample sizes (AIC_C_; Burnham & Anderson, 2002) to determine the best fitting detection function for the data. We estimated density as a derived parameter from the top AIC_C_-ranked models. Models assumed that individuals were identified correctly, populations were demographically closed during sampling, and detections were independent conditional on activity center (Borchers & Efford, 2008; Efford, 2004). We evaluated support for the following sources of variation in *g0* and $\sigma$ by comparing AIC_C_ values among candidate models: (1) four possible behavioural responses – individual detection probability increases or decreases at a particular surveyed area depending on detection at previous occasion(s) (*bk*); individual detection probability increases or decreases at a particular surveyed area depending on detection at the immediate preceding occasion (*Bk*); detection probability of a particular surveyed area depends on whether an individual was detected there at previous occasion(s) (*k*); detection probability of a particular surveyed area depends on whether an individual was detected there at the immediately preceding occasion (*K*); and (2) two possible time effects, with detection varying with the sampling occasion (*t*) or whether there was a tendency for higher or lower individual detectability along the course of the study (*T*; Borchers & Efford, 2008; Efford & Mowat, 2014).

We assumed detections to be independent between individuals; violating this assumption can lead to overdispersion, which does not affect the point estimates of density or model parameters, but can lead to underestimated variance, and over-parameterized models to be selected by AIC_c_ (Anderson, Burnham, & White, 1994; Borchers & Efford, 2008).

## References

Anderson D. R., Burnham K. B., White GC (1994) Model selection in overdispersed capture-recapture data. *Ecology*, **75(6)**, 1780–1793. Doi:[10.2307/1939637](https://doi.org/10.2307/1939637)

Borchers DL, Efford MG (2008) Spatially explicit maximum likelihood methods for capture-recapture studies. *Biometrics*, **64**, 377–385. Doi:[10.1111/j.1541-0420.2007.00927.x](https://doi.org/10.1111/j.1541-0420.2007.00927.x)

Burnham KP, Anderson DR (2002) *Model selection and multimodel inference: A practical information-theoretic approach*. Springer, New York, NY.

COSEWIC (2002) *COSEWIC assessment and update status report on the woodland caribou Rangifer tarandus caribou in Canada*. Committee on the Status of Endangered Wildlife in Canada, Ottawa.

Courtois R, Bernatchez L, Ouellet JP, Breton L (2003) Significance of caribou (*Rangifer tarandus*) ecotypes from a molecular genetics viewpoint. *Conservation Genetics*, **4**, 393–404. Doi:[https://doi.org/10.1023/a:1024033500799](https://doi.org/https://doi.org/10.1023/a:1024033500799)

Efford M (2004) Density estimation in live-trapping studies. *Oikos*, **106**, 598–610. Doi :[10.1111/j.0030-1299.2004.13043.x](https://doi.org/10.1111/j.0030-1299.2004.13043.x)

Efford MG (2011) Estimation of population density by spatially explicit capture-recapture analysis of data from area searches. *Ecology*, **92**, 2202–2207. Doi:[10.1890/11-0332.1](https://doi.org/10.1890/11-0332.1)

Efford MG, Mowat G (2014) Compensatory heterogeneity in spatially explicit capture-recapture data. *Ecology*, **95**, 1341–1348. Doi:[10.1890/13-1497.1](https://doi.org/10.1890/13-1497.1)

Environment Canada. (2011). *Scientific assessment to inform the identification of critical habitat for woodland caribou (Rangifer tarandus caribou), boreal population, in Canada: 2011 update* (p. 102 plus appendices). Ottawa, Ontario, Canada.

Environment Canada. (2012). *Recovery strategy for the woodland caribou (Rangifer tarandus caribou), boreal population, in Canada. Species at Risk Act recovery strategy series* (p. xi + 138). Ottawa, Ontario, Canada.

Festa-Bianchet, M., Ray, J. C., Boutin, S., Côté, S. D., & Gunn, A. (2011). Conservation of caribou (*Rangifer tarandus*) in Canada: An uncertain future. *Canadian Journal of Zoology*, *89*(5), 419–434. Doi:[10.1139/z11-025](https://doi.org/10.1139/z11-025)

Graham K, Stenhouse G (2014) Home range, movements, and denning chronology of the grizzly bear (*Ursus arctos*) in West-Central Alberta. *The Canadian Field-Naturalist*, **128**, 223–234. Doi:[10.22621/cfn.v128i3.1600](https://doi.org/10.22621/cfn.v128i3.1600)

Government of Alberta (2017) *DRAFT provincial woodland caribou range plan*. Retrieved from <http://aep.alberta.ca/fish-wildlife/wildlife-management/caribou-range-planning/>

Hervieux, D., Hebblewhite, M., DeCesare, N. J., Russell, M., Smith, K., Robertson, S., & Boutin, S. (2013). Widespread declines in woodland caribou (*Rangifer tarandus caribou*) continue in Alberta. *Canadian Journal of Zoology*, *91*(12), 872–882. Doi:[10.1139/cjz-2013-0123](https://doi.org/10.1139/cjz-2013-0123)

James, A. R. C., & Stuart-Smith, A. K. (2000). Distribution of caribou and wolves in relation to linear corridors. *Journal of Wildlife Management*, *64*(1), 154–159. Doi:[10.2307/3802985](https://doi.org/10.2307/3802985)

Lamb, C. T., Mowat, G., Reid, A., Smit, L., Proctor, M., McLellan, B. N., … Boutin, S. (2018). Effects of habitat quality and access management on the density of a recovering grizzly bear population. *Journal of Applied Ecology*, *55*(3), 1406–1417. Doi:[10.1111/1365-2664.13056](https://doi.org/10.1111/1365-2664.13056)

Rettie, W. J., & Messier, F. (2001). Range use and movement rates of woodland caribou in askatchewan. *Canadian Journal of Zoology*, *79*(11), 1933–1940. Journal Article. Doi:[10.1139/cjz-79-11-1933](https://doi.org/10.1139/cjz-79-11-1933)

Royle, J. A., & Young, K. V. (2008). A hierarchical model for spatial capture-recapture data. *Ecology*, *89*(8), 2281–2289. Doi:[10.1890/07-0601.1](https://doi.org/10.1890/07-0601.1)

Schaefer, J. A. (2003). Long-term range recession and the persistence of caribou in the taiga. *Conservation Biology*, *17*(5), 1435–1439. Doi:[10.1046/j.1523-1739.2003.02288.x](https://doi.org/10.1046/j.1523-1739.2003.02288.x)

Smith, K. G., Ficht, E. J., Hobson, D., Sorensen, T. C., & Hervieux, D. (2000). Winter distribution of woodland caribou in relation to clear-cut logging in west-central Alberta. *Canadian Journal of Zoology*, *78*(8), 1433–1440. Doi:[10.1139/cjz-78-8-1433](https://doi.org/10.1139/cjz-78-8-1433)

Stuart-Smith, A. K., Bradshaw, C. J. A., Boutin, S., Hebert, D. M., & Rippin, A. B. (1997). Woodland caribou relative to landscape patterns in northeastern Alberta. *The Journal of Wildlife Management*, *61*(3), 622–633. Doi:[10.2307/3802170](https://doi.org/10.2307/3802170)

Sun, C. C., Fuller, A. K., & Royle, J. A. (2014). Trap configuration and spacing influences parameter estimates in spatial capture-recapture models. *PLOS ONE*, *9*(2), e88025. Doi:[10.1371/journal.pone.0088025](https://doi.org/10.1371/journal.pone.0088025)

Whittington, J., & Sawaya, M. A. (2015). A comparison of grizzly bear demographic parameters estimated from non-spatial and spatial open population capture-recapture models. *Plos One*, *10*(7), 17. Doi:[10.1371/journal.pone.0134446](https://doi.org/10.1371/journal.pone.0134446)

Wilton, C. M., Puckett, E. E., Beringer, J., Gardner, B., Eggert, L. S., & Belant, J. L. (2014). Trap array configuration influences estimates and precision of black bear density and abundance. *Plos One*, *9*(10), 10. Journal Article. Doi :[10.1371/journal.pone.0111257](https://doi.org/10.1371/journal.pone.0111257)

##### Appendix 2 : Inhomogeneous population simulations

Table S2.1: Simulated population parameters used in clustering simulations.

| **Population** | **Range Size (km²)** | **Density (ha)** | **g0** | **σ** | **Detector spacing** | **n occasions** | **n runs** |
| --- | --- | --- | --- | --- | --- | --- | --- |
| Little Smoky | 3,027 | 0.00030 | 0.030 | 1600 | 1,500 | 3 | 100 |
| Cold Lake | 7,108 | 0.00060 | 0.015 | 3400 | 1,500 | 3 | 100 |
| Slave Lake | 1,485 | 0.00025 | 0.250 | 1200 | 1,500 | 3 | 100 |

**Little Smoky**


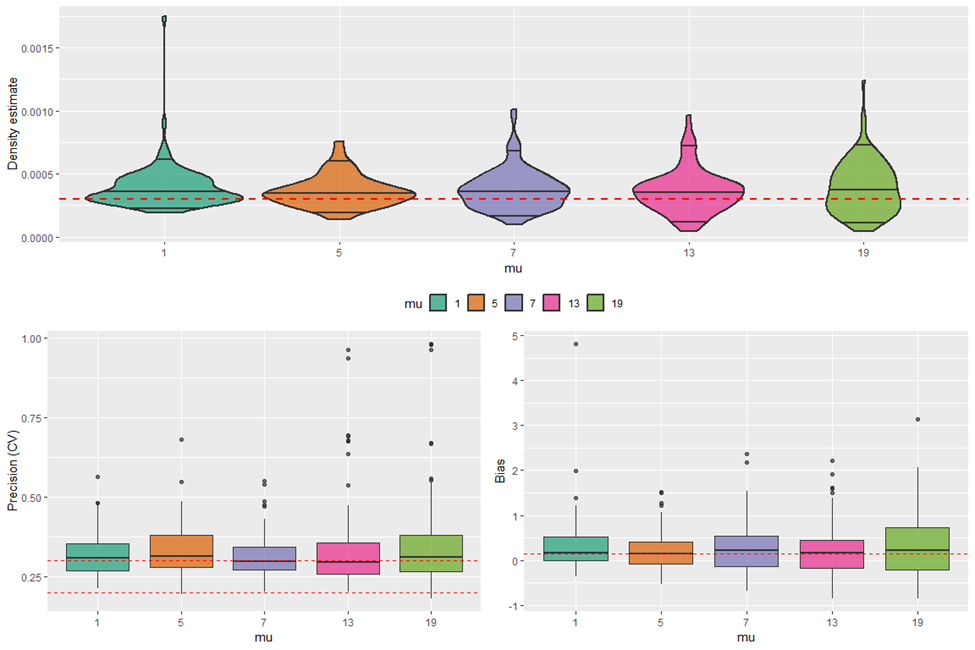


Figure S2.1: Little Smoky simulation results. Top shows the distribution of simulated density estimates for each level of clustering (mu), with horizontal dotted line representing the initial density. Bottom left shows distribution of precision (CV), with horizontal dotted lines representing 20% and 30% CV. Bottom right graph shows distribution of relative bias, with the horizontal dotted line representing 20% bias.

**Cold Lake**


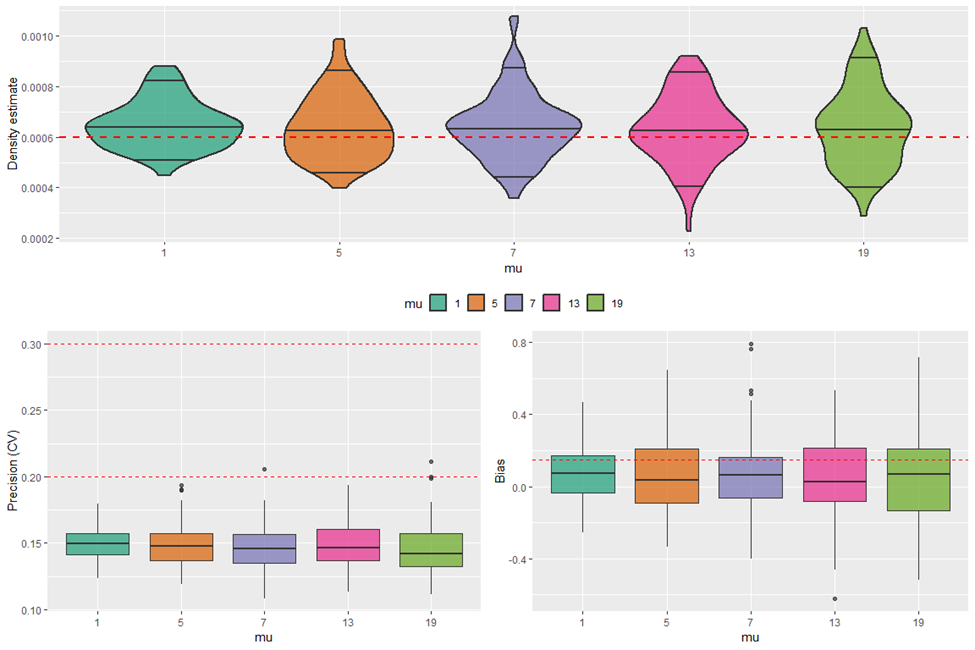


Figure S2.2: Cold Lake simulation results. Top shows the distribution of simulated density estimates for each level of clustering (mu), with horizontal dotted line representing the initial density. Bottom left shows distribution of precision (CV), with horizontal dotted lines representing 20% and 30% CV. Bottom right graph shows distribution of relative bias, with the horizontal dotted line representing 20% bias.

**Slave Lake**


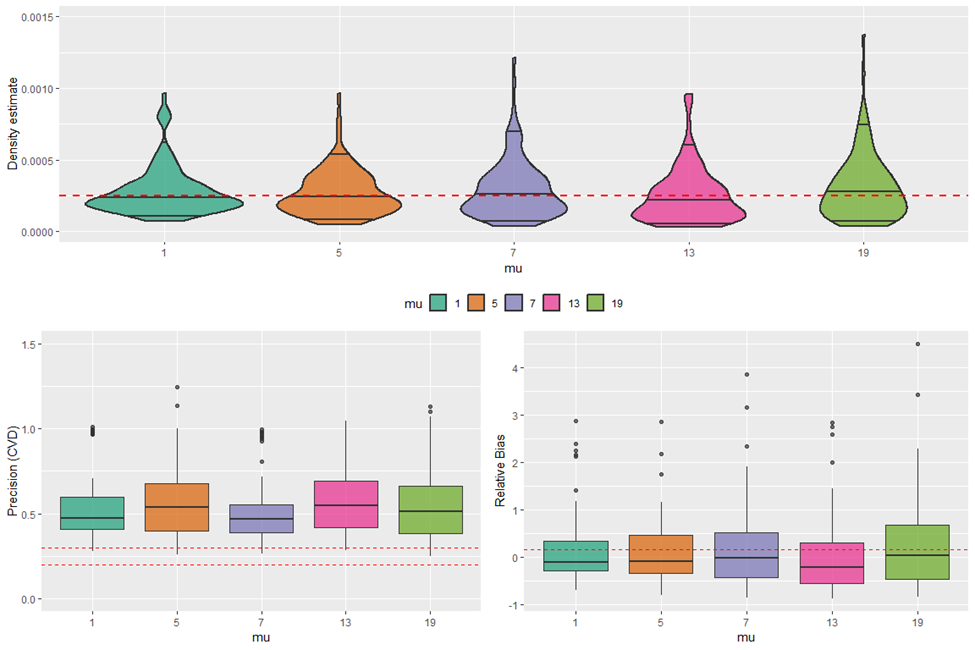


Figure S2.3: Slave Lake simulation results. Top shows the distribution of simulated density estimates for each level of clustering (mu), with horizontal dotted line representing the initial density. Bottom left shows distribution of precision (CV), with horizontal dotted lines representing 20% and 30% CV. Bottom right graph shows distribution of relative bias, with the horizontal dotted line representing 20% bias.

##### Appendix 3: SCR model results and correlation results

**Both Sexes**

Table S3.1: Both sexes capture data

| **Population** | **Number of Individuals** | **Number of Captures** | **Number of Recaptures** | **Number of Spatial Recaptures** |
| --- | --- | --- | --- | --- |
| Cold Lake | 261 | 416 | 155 | 148 |
| ESAR | 400 | 606 | 206 | 188 |
| Little Smoky | 69 | 105 | 36 | 36 |
| Nipisi | 62 | 138 | 76 | 72 |
| Red Earth | 388 | 931 | 543 | 530 |
| Slave Lake | 41 | 85 | 44 | 38 |
| WSAR | 489 | 805 | 316 | 314 |
| Total | 1710 | 3086 | 1376 | 1326 |

**Females**

Table S3.2: Female capture data

| **Population** | **Number of Individuals** | **Number of Captures** | **Number of Recaptures** | **Number of Spatial Recaptures** |
| --- | --- | --- | --- | --- |
| Cold Lake | 172 | 267 | 95 | 92 |
| ESAR | 280 | 413 | 133 | 123 |
| Little Smoky | 46 | 68 | 22 | 22 |
| Nipisi | 40 | 82 | 42 | 39 |
| Red Earth | 223 | 539 | 316 | 307 |
| Slave Lake | 28 | 61 | 33 | 31 |
| WSAR | 316 | 511 | 195 | 193 |
| Total | 1105 | 1941 | 836 | 807 |

Table S3.3: Spatially-explicit capture-recapture density estimates for female boreal caribou in Alberta, Canada. Density estimates (D) are per 1000 km², SE(D) is the standard error of the density estimate, CV(D) is the coefficient of variation (SE of density estimate/density estimate), g0 indicates the capture probability at the home range center, sigma is the spatial scale parameter in meters, and N is the abundance over the study area.

| **Population** | **D (95% CI)** | **SE(D)** | **CV(D)** | **g0 (95% CI)** | **σ (95% CI)** | **N (95% CI)** |
| --- | --- | --- | --- | --- | --- | --- |
| Cold Lake | 36.7 (29.6-45.5) | 4.03 | 0.11 | 0.023 (0.011-0.048) | 2593 (1877-3649) | 261 (210-323) |
| ESAR | 37.6 (30.6-46.1) | 3.93 | 0.10 | 0.022 (0.013-0.038) | 1874 (1500-2342) | 481 (392-590) |
| Little Smoky | 21.7 (14.6-32.4) | 4.46 | 0.20 | 0.008 (0.002-0.028) | 3085 (1764-5396) | 66 (44-98) |
| Nipisi | 20.8 (14.2-30.4) | 4.07 | 0.20 | 0.048 (0.02-0.115) | 2091 (1393-3145) | 43 (29-62) |
| Red Earth | 9.2 (8-10.6) | 0.66 | 0.07 | 0.028 (0.019-0.041) | 2928 (2485-3449) | 221 (193-255) |
| Slave Lake | 14.8 (9.7-22.7) | 3.25 | 0.22 | 0.422 (0.1-1.824) | 1134 (703-1842) | 22 (14-34) |
| WSAR | 27.9 (24.1-32.3) | 2.10 | 0.08 | 0.038 (0.026-0.056) | 2679 (2155-3334) | 428 (369-496) |


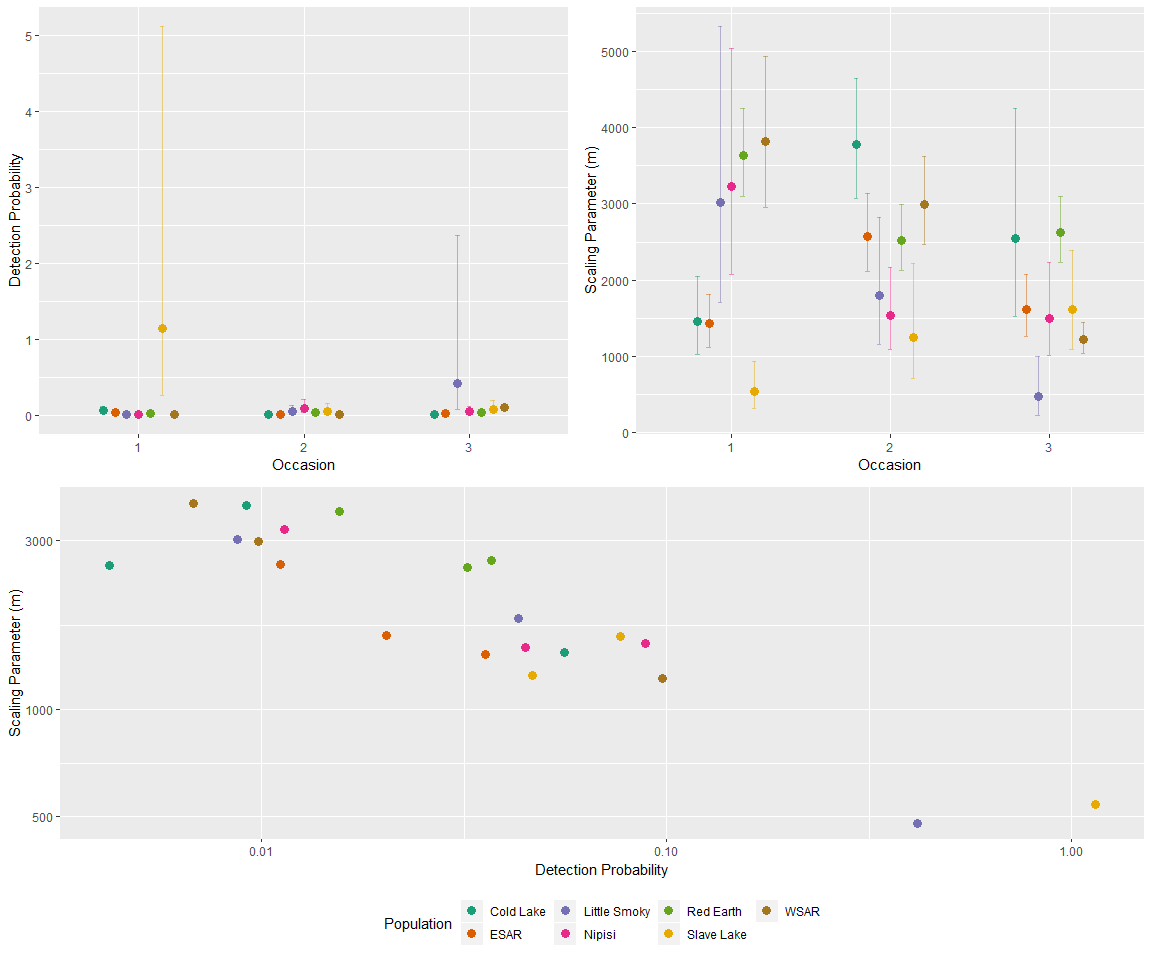


Figure S3.1: Female detection probability (*g0*) and scaling parameter (σ) by population.

**Males**

Table S3.4: Male capture data

| **Population** | **Number of Individuals** | **Number of Captures** | **Number of Recaptures** | **Number of Spatial Recaptures** |
| --- | --- | --- | --- | --- |
| Cold Lake | 89 | 149 | 60 | 56 |
| ESAR | 120 | 193 | 73 | 65 |
| Little Smoky | 23 | 37 | 14 | 14 |
| Nipisi | 22 | 56 | 34 | 33 |
| Red Earth | 165 | 392 | 227 | 222 |
| Slave Lake | 13 | 24 | 11 | 7 |
| WSAR | 173 | 294 | 121 | 121 |
| Total | 605 | 1145 | 540 | 518 |

Table S3.5: Spatially-explicit capture-recapture density estimates for male boreal caribou in Alberta, Canada. Density estimates (D) are per 1000 km², SE(D) is the standard error of the density estimate, CV(D) is the coefficient of variation (SE of density estimate/density estimate), g0 indicates the capture probability at the home range center, sigma is the spatial scale parameter in meters, and N is the abundance over the study area.

| **Population** | **D (95% CI)** | **SE(D)** | **CV(D)** | **g0 (95% CI)** | **σ (95% CI)** | **N (95% CI)** |
| --- | --- | --- | --- | --- | --- | --- |
| Cold Lake | 15.6 (12-20.1) | 2.05 | 0.13 | 0.053 (0.024-0.12) | 1788 (1310-2444) | 111 (86-143) |
| ESAR | 11.9 (9.3-15.1) | 1.48 | 0.12 | 0.045 (0.031-0.065) | 1506 (1298-1748) | 152 (119-194) |
| Little Smoky | 9.1 (5.5-15) | 2.37 | 0.26 | 0.051 (0.021-0.122) | 1002 (600-1673) | 28 (17-46) |
| Nipisi | 9.9 (6.3-15.6) | 2.32 | 0.23 | 0.074 (0.036-0.156) | 1607 (1266-2040) | 20 (13-32) |
| Red Earth | 6.8 (5.8-8) | 0.57 | 0.08 | 0.031 (0.02-0.02) | 2477 (2047-2998) | 163 (138-192) |
| Slave Lake | 15.6 (5.8-42.2) | 8.44 | 0.54 | 0.023 (0.005-0.106) | 1558 (925-2626) | 23 (9-63) |
| WSAR | 13.4 (11.1-16.1) | 1.26 | 0.09 | 0.024 (0.013-0.045) | 3347 (2592-4321) | 205 (171-247) |


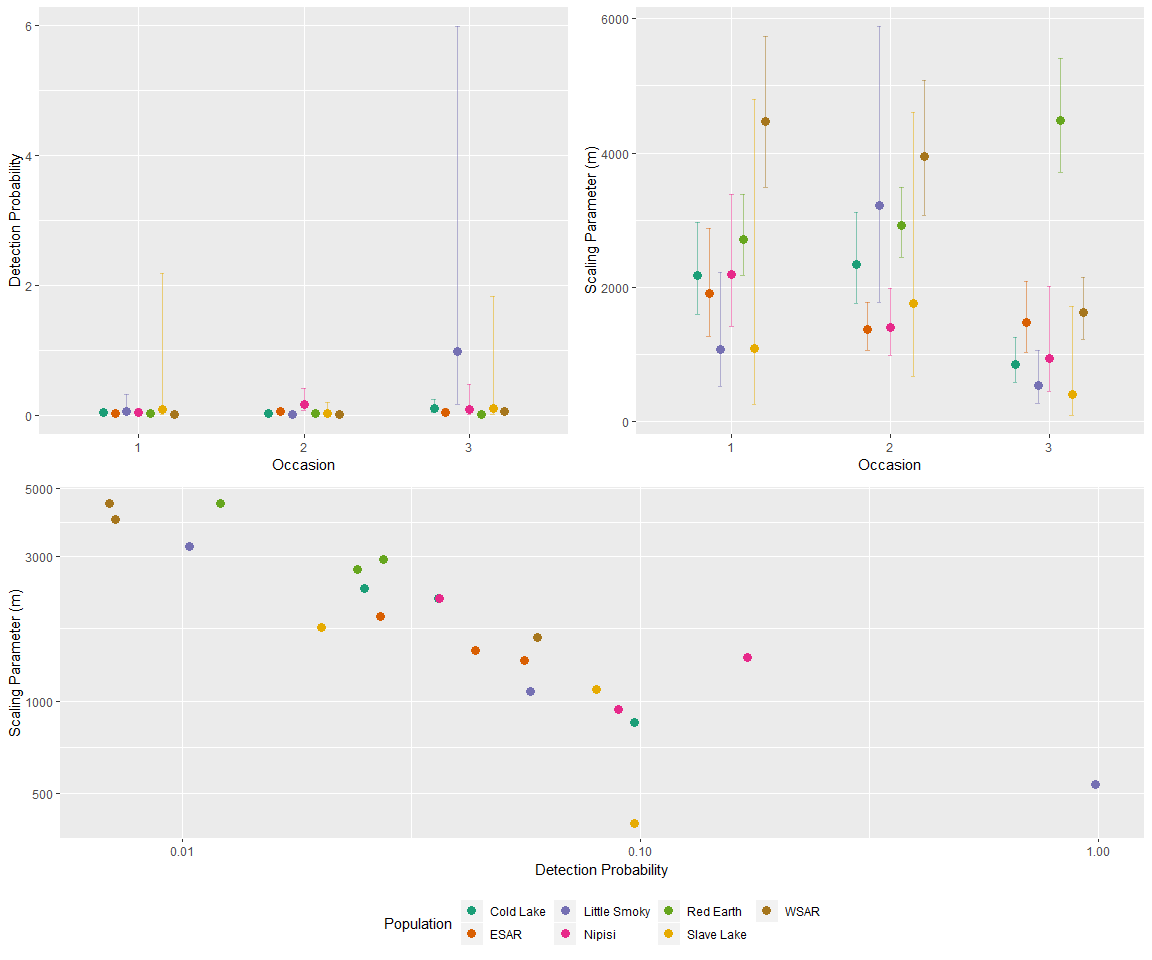


Figure S3.2: Male detection probability (*g0*) and scaling parameter (σ) by population.

**Correlation Results**

Table S3.6: Correlation results (r2) between the number of individuals and precision (CV(D)) and relative bias (RB) for each population and sex. Significance level (p-value) in brackets.

| **Population** | **Sex** | **CV(D)** | **RB** |
| --- | --- | --- | --- |
| Cold Lake | Both Sexes | -0.758 (0.00428) | -0.21 (0.535) |
| ESAR | Both Sexes | -0.791 (0.00217) | -0.21 (0.535) |
| Little Smoky | Both Sexes | -0.729 (0.0167) | -0.429 (0.249) |
| Nipisi | Both Sexes | -0.856 (0.000383) | -0.646 (0.0318) |
| Red Earth | Both Sexes | -0.963 (8.13e-06) | 0.0389 (0.921) |
| Slave Lake | Both Sexes | -0.00178 (0.997) | 0.289 (0.53) |
| WSAR | Both Sexes | -0.866 (0.000271) | -0.351 (0.29) |
| Cold Lake | Female | -0.576 (0.05) | -0.187 (0.582) |
| ESAR | Female | -0.766 (0.00369) | -0.459 (0.156) |
| Little Smoky | Female | -0.736 (0.0152) | -0.0597 (0.879) |
| Nipisi | Female | -0.808 (0.00147) | -0.603 (0.0494) |
| Red Earth | Female | -0.903 (5.7e-05) | -0.754 (0.00736) |
| Slave Lake | Female | -0.745 (0.0338) | 0.519 (0.232) |
| WSAR | Female | -0.854 (0.000402) | -0.322 (0.335) |
| Cold Lake | Male | -0.718 (0.0086) | -0.513 (0.107) |
| ESAR | Male | -0.857 (0.000366) | -0.368 (0.266) |
| Little Smoky | Male | -0.386 (0.393) | -0.794 (0.0592) |
| Nipisi | Male | -0.918 (2.59e-05) | -0.636 (0.0354) |
| Red Earth | Male | -0.921 (2.18e-05) | -0.45 (0.165) |
| Slave Lake | Male | -0.903 (0.0136) | -0.283 (0.644) |
| WSAR | Male | -0.821 (0.00106) | 0.153 (0.653) |

Table S3.7: Correlation results (r2) between the number of recaptures and precision (CV(D)) and relative bias (RB) for each population and sex. Significance level (p-value) in brackets.

| **Population** | **Sex** | **CV(D)** | **RB** |
| --- | --- | --- | --- |
| Cold Lake | Both Sexes | -0.654 (0.0211) | -0.234 (0.489) |
| ESAR | Both Sexes | -0.717 (0.00875) | -0.278 (0.407) |
| Little Smoky | Both Sexes | -0.671 (0.0338) | -0.428 (0.251) |
| Nipisi | Both Sexes | -0.693 (0.0125) | -0.667 (0.0249) |
| Red Earth | Both Sexes | -0.859 (0.00146) | -0.208 (0.591) |
| Slave Lake | Both Sexes | -0.62 (0.101) | 0.158 (0.735) |
| WSAR | Both Sexes | -0.847 (0.000502) | -0.174 (0.609) |
| Cold Lake | Female | -0.6 (0.0393) | -0.322 (0.335) |
| ESAR | Female | -0.682 (0.0145) | -0.452 (0.162) |
| Little Smoky | Female | -0.647 (0.0432) | -0.349 (0.357) |
| Nipisi | Female | -0.687 (0.0137) | -0.686 (0.0198) |
| Red Earth | Female | -0.79 (0.00226) | -0.689 (0.0191) |
| Slave Lake | Female | -0.873 (0.0046) | 0.0834 (0.859) |
| WSAR | Female | -0.827 (0.000906) | -0.299 (0.372) |
| Cold Lake | Male | -0.622 (0.0308) | -0.431 (0.186) |
| ESAR | Male | -0.823 (0.00101) | -0.391 (0.234) |
| Little Smoky | Male | -0.628 (0.131) | -0.302 (0.56) |
| Nipisi | Male | -0.722 (0.00795) | -0.682 (0.0207) |
| Red Earth | Male | -0.878 (0.000171) | -0.408 (0.213) |
| Slave Lake | Male | -0.594 (0.214) | -0.244 (0.692) |
| WSAR | Male | -0.803 (0.00166) | 0.211 (0.534) |

Table S3.8: Correlation results (r2) between the number of spatial recaptures and precision (CV(D)) and relative bias (RB) for each population and sex. Significance level (p-value) in brackets.

| **Population** | **Sex** | **CV(D)** | **RB** |
| --- | --- | --- | --- |
| Cold Lake | Both Sexes | -0.653 (0.0213) | -0.228 (0.5) |
| ESAR | Both Sexes | -0.72 (0.00832) | -0.235 (0.488) |
| Little Smoky | Both Sexes | -0.671 (0.0338) | -0.428 (0.251) |
| Nipisi | Both Sexes | -0.704 (0.0106) | -0.678 (0.0219) |
| Red Earth | Both Sexes | -0.861 (0.00139) | -0.208 (0.591) |
| Slave Lake | Both Sexes | -0.615 (0.105) | 0.0571 (0.903) |
| WSAR | Both Sexes | -0.845 (0.000547) | -0.179 (0.599) |
| Cold Lake | Female | -0.595 (0.0411) | -0.314 (0.347) |
| ESAR | Female | -0.685 (0.014) | -0.442 (0.173) |
| Little Smoky | Female | -0.647 (0.0432) | -0.349 (0.357) |
| Nipisi | Female | -0.704 (0.0106) | -0.703 (0.0159) |
| Red Earth | Female | -0.789 (0.00226) | -0.684 (0.0203) |
| Slave Lake | Female | -0.882 (0.00377) | 0.00505 (0.991) |
| WSAR | Female | -0.826 (0.000921) | -0.289 (0.389) |
| Cold Lake | Male | -0.623 (0.0306) | -0.422 (0.196) |
| ESAR | Male | -0.83 (0.000829) | -0.355 (0.285) |
| Little Smoky | Male | -0.628 (0.131) | -0.302 (0.56) |
| Nipisi | Male | -0.729 (0.00713) | -0.688 (0.0193) |
| Red Earth | Male | -0.879 (0.000163) | -0.409 (0.212) |
| Slave Lake | Male | -0.523 (0.287) | 0.125 (0.841) |
| WSAR | Male | -0.799 (0.00184) | 0.194 (0.568) |

##### Appendix 4: Comparing different discretize spacings

These tests were run on Little Smoky. Plotting the results of the top models from each spacing. As we increase the discretize spacing, we are increasing the 95% confidence intervals. Males performed similarly from 750 m to 2250 m but were inflated at 2500m. Females performed similarly from 750 m to 1500 m, but greater than 1500 m the estimates were inflated.


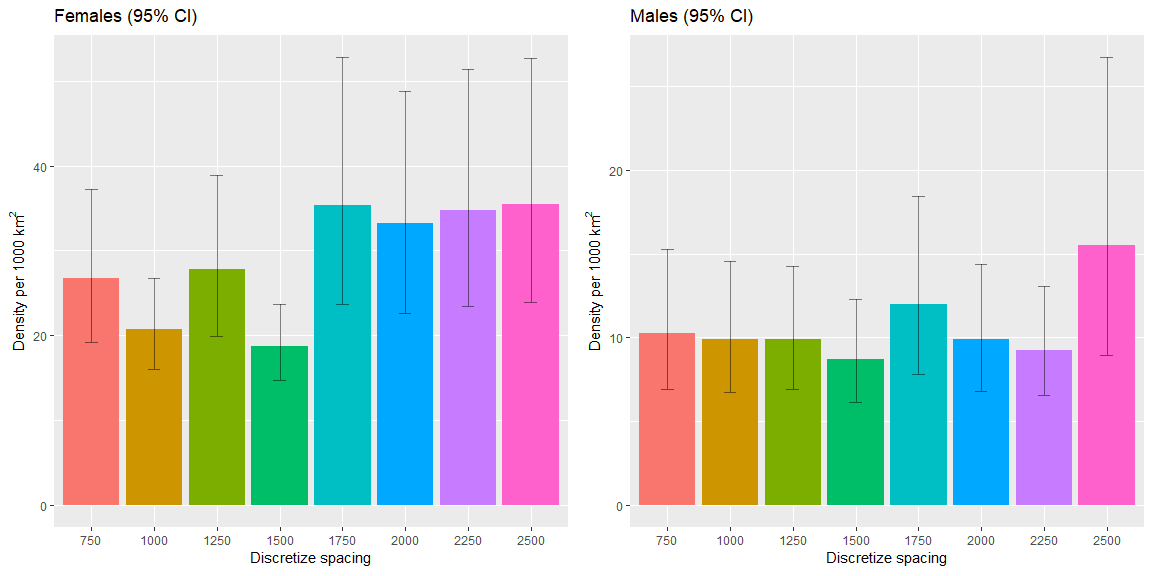


Figure S4.1: Resulting density estimates from various discretize spacing widths for females and males.

All models were run at 1500m discretize spacing. Originally 2500m was used for the three populations (Little Smoky, A la Peche and Cold Lake), but 1500m was selected as the best spacing. Transect lines were flown 3000m apart; it is likely that choosing a spacing of 2500m was incorrect as it did not reflect the flight transect spacings. Selecting a discretize spacing of 1500m represents half the width of the flight transects.
